# Surface Activation of Calcium Zirconate-Calcium Stabilized Zirconia Eutectic Ceramics with Bioactive Wollastonite-Tricalcium Phosphate Coatings

**DOI:** 10.3390/jfb14100510

**Published:** 2023-10-11

**Authors:** Daniel Sola, Eloy Chueca, Shunheng Wang, José Ignacio Peña

**Affiliations:** 1 Instituto de Nanociencia y Materiales de Aragón, Universidad de Zaragoza-CSIC, 50018 Zaragoza, Spain; 2 Aragonese Foundation for Research and Development (ARAID), 50018 Zaragoza, Spain; 3 Centro de Investigación en Óptica y Nanofísica, Campus Espinardo, Universidad de Murcia, 30100 Murcia, Spain; 4 School of Materials Science and Engineering, Tiangong University, Tianjin 300387, China

**Keywords:** laser floating zone, directionally solidified ceramic eutectics, calcium zirconate, zirconium oxide, bioactive glasses, bone implant

## Abstract

In this work, we have developed and characterized a ceramic composite based on a core of directionally solidified calcium zirconate-calcium stabilized zirconia (CZO-CSZ) eutectic composite coated with a bioactive glass-ceramic. The aim is to research new orthopedic implants as an alternative to conventional 3Y-TZP bioinert ceramics. The CZO-CSZ eutectic rods were grown from the melt of rods of CaO-ZrO_2_ in the eutectic composition using the laser floating zone technique (LFZ). The mechanical results indicated that directional eutectics prepared with this technique exhibited good mechanical strength and significant hardness and toughness. The LFZ technique was also used to melt the bioactive coating previously placed by dip coating on the CZO-CSZ rod surface. Depending on the thickness of the coating and the applied laser power, an alloying or coating process was achieved. In the first case, the coating was diluted with the surface of the eutectic cylinder, leading to the segregation of the calcium zirconate and zirconia phases and the formation of a bioactive phase embedding zirconia particles. In the second case, a layer of ceramic glass was formed, well attached to the eutectic cylinder. These layers were both studied from the microstructural and bioactivity points of view.

## 1. Introduction

Ceramics and glasses are often used as bone substitutes because of their chemical compatibility and/or similar mineral composition with bones. However, in clinical applications, there are some difficulties related to the brittleness and low reliability of the mechanical behavior of ceramics. Therefore, it is meaningful to search for ceramics with good mechanical properties that also possess bioactive properties. ZrO_2_-based materials have excellent properties because of their toughness and resistance, better than other bioceramic materials. Along with their good biocompatibility, zirconia materials find many applications in bone surgery and dental applications [1,2,3]. Eutectic ceramics are arousing considerable interest due to their good mechanical behavior, but they should be enhanced by reducing aging sensitivity and improving osteointegration performance [4,5].

The main advantage of directionally solidified eutectic composites (DSECs) for structural applications is that they are very stable, even at high temperatures. This is because their microstructures are produced directly from the molten state under conditions of thermodynamic equilibrium, which gives them great steadiness. Furthermore, the phase interfaces are strong and clean, the grain boundaries, if present, are relatively few in number, and after solidification, the samples almost reach the theoretical density. In addition, an adequate selection of phases makes it possible to obtain very resistant and tough materials, enhanced by the presence of a fine and uniform microstructure. In fact, the presence of finely dispersed fibrous or lamellar phases can serve to deflect or hinder crack propagation by absorbing energy during fracture.

The eutectic composite CaZrO_3_-ZrO_2_(CaO) has been studied for its optical properties [6,7] and electric conductivity at high temperatures [8]. However, as a structural material, it has not been studied in detail, despite the fact that it presents some interesting aspects. The eutectic microstructure consists of alternate lamellae of CaZrO_3_ (CZO) and Ca-doped ZrO_2_ (CSZ). Approximately 25 mol% of the ZrO_2_ phase is replaced by CaO, stabilizing the ZrO_2_ in its cubic form with a fluorite structure. This cubic structure is preserved after cooling to room temperature. This avoids the weakening effects due to the presence of a monoclinic phase, so that degradation due to aging in a humid environment does not occur in this material. The CaZrO_3_ phase crystallizes in an orthorhombic Pnma perovskite structure, and because of the perfect thermal expansion match between the phases, improved mechanical properties may be observed as well as a high resistance to thermal shock [9]. The number and size of the microcracks are significantly limited upon cooling, even under high thermal gradients such as those present in the laser floating zone technique used in this work.

In biomedical applications, the use of coatings is very interesting because they provide improved surface properties while maintaining favorable bulk properties. The bioactivity of Al_2_O_3_/ZrO_2_ eutectics was enhanced by the coating of the Ca-P phase by laser cladding [4]. Laser cladding has been widely used for the preparation of bioceramic coatings on the surface of metals with appropriate biological activity [10,11] or other bioinert ceramics [4]. In these studies, good strength and biological activity were achieved by controlling the composition and microstructure of the coating and optimizing the laser-processing parameters.

In this study, the laser floating zone (LFZ) was utilized to grow rods of CaO-ZrO_2_ in the eutectic composition. The assessment of how the microstructure was related to the solidification rate was performed. These rods were laser-cladded with a bioceramic based on CaSiO_3_ (Wollastonite, W) and Ca_3_(PO_4_)_2_ (tricalcium phosphate, TCP) in the eutectic composition. W-TCP bioeutectic can be grown by LFZ melting as ceramic or glass, depending on the growth conditions [4]. They both have shown the capability to generate a hydroxyapatite (Hap) layer in simulated biological conditions (SBFs) and have been applied for bone repair, as reported by De Aza [12,13,14].

In our work, the laser cladding process consisted of the melting of the bioceramic coating with a controlled dilution of the eutectic nucleus, ensuring good adhesion between both materials.

## 2. Materials and Methods

The following reagents were used for the preparation of the samples used in this study: zirconium oxide (Aldrich, 99%, Munich, Germany), calcium oxide (Aldrich, 99.9%, Munich, Germany), calcium silicate (Aldrich, 99%, Munich, Germany), tricalcium phosphate (Carlo Erba Reagenti, Barcelona, Spain), ethanol (PanReac, 99.8%, Barcelona, Spain), and Beycostat C213 (CECA, Montluçon, France).

A mixture of CaO and ZrO_2_ powders with binary eutectic composition (40 mol% CaO-60 mol% ZrO_2_ nominal) was pressed inside an isostatic press (MEGA, Berriz, Spain) using a rubber tube for 3 min at 200 MPa, yielding cylinders from 7 to 10 cm long and about 3 mm in diameter, which were subsequently densified by sintering at 1500 °C for 12 h.

These cylinders were directionally solidified by the LFZ technique previously described [15]. The LFZ growth was initiated by first melting the tip of the upper cylinder, used as a seed, and inserting the top of the lower cylinder (precursor) into this molten volume. Once the molten zone was established as a liquid bridge between the two rods, they were moved at specified rates. A stable directional growth was achieved by adjusting the laser power input to maintain the molten zone length about 1.5 times the rod diameter. In the first step, the rods moved upward at rates of 200 mm/h with a counterrotation of 50 rpm, increasing the densification and improving the alignment of the precursor rod. The second step of growth was performed downward to separate the bubbles present in the molten zone aside from the solidification front, with counterrotation of 50 rpm or without rotation when phase segregation was desirable to be avoided.

W-TCP coatings were prepared on the surface of CZO-CSZ rods by the dip coating technique, dipping and withdrawing the rods at a constant rate in a ceramic solution. The composition of the solution was (in wt%): 42.37 W-TCP; 44.49 ethanol; 0.42 Beycostat C213 as the dispersant agent; and 12.72 PVB (polyvinyl butyral) as the binder. The number of dips and the adjustment of the concentration of the solution allowed for controlling the thickness of the layer. Wollastonite (W) and tricalcium phosphate (TCP) eutectic was previously prepared following the phase diagram of calcium silicate (CS)–Tricalcium phosphate (TCP) reported by De Aza et al. [16], where the eutectic point was placed at 80–20 mol% of CaSiO_3_ and 20 Ca_3_(PO_4_)_2_, respectively.

The coated cylinders were sintered at 1200 °C and then superficially melted using the LFZ technique. The samples were moved downward at a rate of 100 mm/h. To homogenize the heat produced in the irradiated area, the sample was simultaneously rotated at 200 rpm. The suitable control of the laser power allowed for melting only the coating and minimizing the dilution with the substrate.

Growing rates from 50 to 500 mm/h were used to evaluate the influence of the processing parameters on the microstructure of CZO-CSZ eutectic crystals. Longitudinal and transversal sections of the rods were examined under electron microscopy (FESEM, model Carl Zeiss MERLIN, Jena, Germany)with an Energy-Dispersive X-ray Spectroscopy (EDS) detector. The composition, spatial distribution, and size of the phases were determined.

A Matsuzawa MXT50 microhardness indenter (Matsuzawa, Tokio, Japan) was utilized to measure the Vickers hardness. At least ten valid microindentations were made in each sample by loading the specimens with 0.2 kgf for 15 s. For the toughness assessment, the load was changed to 1 kgf to induce crack formation. After each indentation, measurements of the size and length of the cracks from the corners of the indentation were performed. The three-point bend test was performed to evaluate the flexure strength by means of an Instron testing machine (model 5565, Instron, Norwood, MA, USA).

In vitro bioactivity of the W-TCP coatings on the CZO-CSZ samples was evaluated by means of the test proposed by Kokubo [17]. Rod samples were cut, and the resulting discs were stored in simulated body fluid (SBF) for four weeks at the temperature of the human body using a stove (Memmert GmbH, model 100–800, Schwabach, Germany).

Micro-Raman characterization was performed by using a homemade micro-Raman microscope, which consisted of an optical microscope coupled to a Raman Spectrometer (SR303i-B, Andor, Belfast, Northern Ireland) equipped with a thermoelectric-cooled CCD detector (Newton 920, Andor, Belfast, Northern Ireland). As the excitation source, a cw-laser source at 532 nm was utilized. Microscope objectives with high numerical apertures (0.85 NA and 0.90 NA) allowed for the collection of the backscattered light. In order to prevent the sample from being locally heated, the output laser power was maintained below 20 mW.

## 3. Results

### 3.1. CZO-CSZ Eutectic

**Figure 1:**
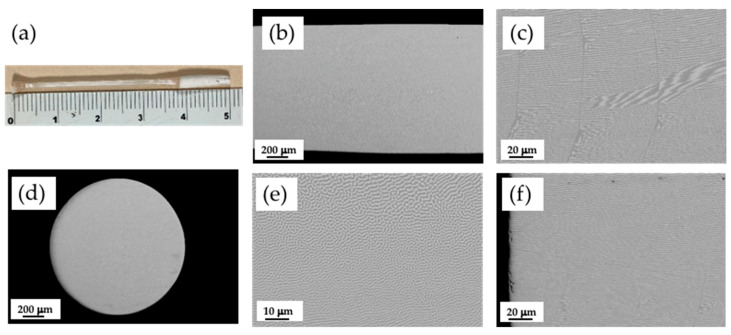
CZO-CSZ eutectic rod grown at 200 mm/h by LFZ (**a**). SEM images of the longitudinal and transversal cross-sections. Longitudinal general view of the sample (**b**) and detailed zone of the center (**c**). Transversal general view (**d**) and detailed zones of the center (**e**) and edge (**f**) of the rod. Dark and clear phases correspond to CZO and CSZ, respectively.

The CZO-CSZ eutectic composite stands out for its high melting point (2300 °C). The solidification was produced with a lamellar distribution of cubic phases with a volume fraction of the minor phase (CSZ) of 41%. The microstructure of the rods grown by the LFZ technique at different growth rates was studied by scanning electron microscopy (SEM). Figure 1 shows a rod grown at 200 mm/h (a) and electronic micrographs of longitudinal and transverse cross-sections of this sample ((b,c) and (d–f), respectively). We observed dark and clear phases of about one micron in width, corresponding to phases with lower and higher electron densities, respectively. An EDS microanalysis of the phases gave the compositions shown in Table 1. The Zr/Ca ratio was 2.88 for the clear phase, which corresponds to calcium-stabilized zirconia, and 0.99 for the dark phase, corresponding to calcium zirconate. These compositions are very close to the ZrO_2_:30 mol% CaO (Ca_0.25_Zr_0.75_O_1.75_, CSZ) and CaZrO_3_ phases, respectively, as predicted by the phase diagram of the ZrO_2_-CaO system for the eutectic constituent. For comparison purposes, the theoretical compositions predicted by the phase diagram are also given in Table 1. Near the surface of the rod, the lamellae were very well ordered, with the phases aligned parallel to the direction of solidification, as can be seen in Figure 1f. In the center of the rod, the alignment of the lamellae was poor, but despite being arranged in a more intricate way, the distribution of the phases maintained homogeneity (Figure 1e). Figure 1c shows an example of the microstructure in a longitudinal section where the lamellae are shown to be interrupted by bands. These ones, about 66 microns apart, corresponded to the advance of the sample (at 200 mm/h) during one sample spin (50 rpm). If necessary, this microstructural feature might be avoided by eliminating the rotation of both the precursor and the cylinder [18]. Due to the high resistance to thermal shock of this material, samples with a diameter up to 1.5 mm were obtained without cracks despite the high radial gradients present in the sample during cooling, as can be observed in Figure 1a,b,d.

In the explored range of growth speeds (R), between 50 and 300 mm/h, the spacing of the lamellar structure (λ) follows Jackson–Hunt’s theory for ideally coupled eutectic growth (λ^2^R = C, with C = 92 µm^3^ s^−1^). The interspacing eutectic rod grown at higher speeds, where the solidification front is not stable, is unusually high. At a growing rate of 500 mm/h, the resulting interspacing is λ = 1.63 µm, which would correspond to a constant of about 350 µm^3^ s^−1^, close to the constant given by Llorca and Orera [19].

Indentation and bending tests were performed to evaluate the mechanical characteristics of these materials. The results are given in Table 2.

The flexural strength is relatively high compared with other ceramics and eutectics based on CaZrO_3_ and ZrO_2_ [19,20,21]. The modified Griffith equation was used to estimate the critical flaw size required to initiate fracture [22]:

σ = 1/Y [E (2γ)/c]^l/2^, (1)

where σ is the fracture strength, Y is the size factor, E is the elastic modulus, γ is the surface energy, and c is the critical flaw size. Considering Y = 2 and assuming γ of an order of 1 J/m^2^ and E = 226 GPa [23,24], the critical flaw that would initiate fracture is about 0.21 µm, smaller than the spacings obtained with this composite (λ = 1.2 µm at 300 mm/h). This fact suggests that the strength is being controlled by microstructure defects produced during solidification, which are more or less insensible to changes in interlamellar spacings and therefore to differences in the solidification speed [25]. The coefficient of thermal expansion is 10 × 10^−6^ °C^−1^ for both phases [26,27]. They also have a similar thermal conductivity of about 2.5 W/km [28,29]. Therefore, a reinforcing effect due to the generation of residual stresses upon cooling is not to be expected, nor is a significant decrease in resistance with temperature.

### 3.2. Alloying of CZO-CSZ Eutectic with W-TCP

**Figure 2:**
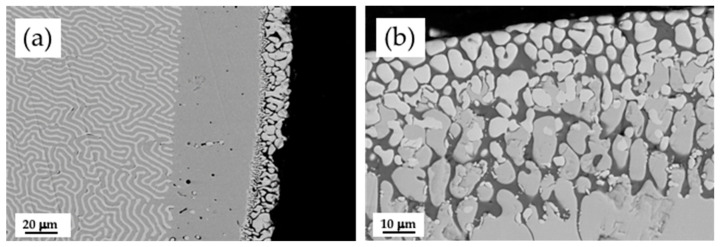
SEM micrographs of a cross-section of a CZO-CSZ rod with a W-TCP coating (**a**). Detail of the microstructure at the surface of the rod (**b**). The composition of the darker phase is close to the W-TCP eutectic.

Rods of CZO-CSZ were covered by dip coating with W-TCP in the eutectic composition. The coating was melted by laser to generate a surface glaze on the cylinder. In Figure 2, the results of the processing are shown. The coating was melted together with the outer zone of the cylinder, producing a segregation of the eutectic phases of the core. In Figure 2a, we can see, from the center of the rod to the edge, three well-differentiated areas: the lamellar eutectic followed by a CZO layer and, in the outer area, particles of CSZ (clear contrast) embedded in a phase with a composition similar to the W-TCP eutectic with a slightly higher calcium content (dark contrast). The theoretical composition (in at %) of the W-TCP eutectic is 60.60 (O), 12.12 (Si), 6.06 (P), and 21.21 (Ca), and the composition of the dark phase embedding the CSZ particles is 59.01 (O), 9.8 (Si), 5.13 (P), and 26.07 (Ca). The higher calcium content with respect to the initial composition suggests that the CZO-CSZ eutectic acts as a source of Ca^2+^ ions. The composite layer of CSZ and W-TCP formed after laser fusion treatment combined both the bioactivity of the bioceramic phase [30,31] and the high strength and toughness of ZrO_2_ [32].

ZrO_2_ was used with hydroxyapatite (HAp) as a reinforcing phase by Htun et al. [33]. They showed that the addition of CaF_2_ and CaO-ZrO_2_ to the HAp matrix produced higher densification and better mechanical properties. The effect of CSZ on HAp was studied by M.F. Vassala [34]. They observed that the addition of 10 wt% of CaZrO_3_ to hydroxyapatite promoted the stabilization of the hydroxyapatite phase at a sintering temperature of 1300 °C, attaining tensile strength and hardness for dense materials of 55 MPa and 6.8 GPa, respectively. The biocomposites maintained good biocompatibility since they favored human osteoblast cell adhesion and proliferation.

**Figure 3:**
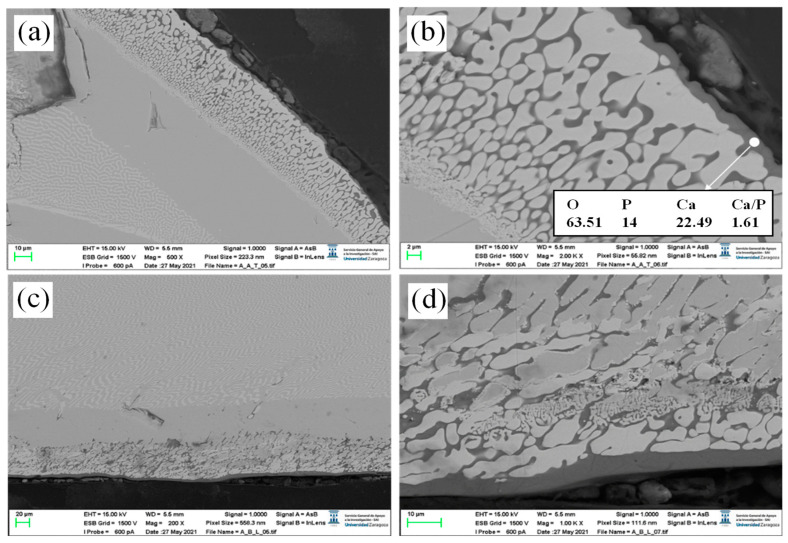
SEM micrographs of transversal (**a**) and longitudinal (**c**) cross-sections of a sample after 4 weeks of soaking in SBF. (**b**,**d**) are detailed zones of the outer part of the rod in contact with the physiological fluid. A layer of hydroxyapatite is formed.

The behavior of this layer in physiological body fluid was explored following the test developed by Kokubo. In Figure 3, the layer formed on the surface of the coated rods after a 4-week immersion period in SBF is shown. Figure 3a,c correspond to the transversal and longitudinal cross-sections of the rod, respectively. A thin layer ranging 2–5 µm thick on the edge of the bioactive coat can be observed. Figure 3b,d are detailed zones of these layers. The composition of the formed layer had a Ca/P ratio of 1.61, close to that of hydroxyapatite (Ca/P = 1.66). Therefore, the presence of the ZrO_2_ phase did not degrade the biocompatibility of the bioactive phase.

Hardness tests were carried out on this sample to evaluate the mechanical properties before and after soaking in SBF. The hardness of the CZO layer in between the CZO-CSZ eutectic and the CZO-W-TCP coating resulted in 6.74 GPa and, as expected, was independent from soaking, which exclusively affected the bioactive coating. In the W-TCP coating, hardness decreased from 4.43 GPa to 2.70 GPa before and after soaking, respectively. The standard deviation in these measurements was estimated at 0.2 GPa. This decrease in hardness is related to the transformation of TCP into HA and the partial dissolution of W in the SBF. A decrease in hardness and the resulting reduction in the elastic modulus may be beneficial for the sample to be used as an implant, as it is required that the elastic modulus of the implant match that of the bone tissue to prevent stress shielding.

### 3.3. Cladding of CZO-CSZ Eutectic with W-TCP

**Figure 4:**
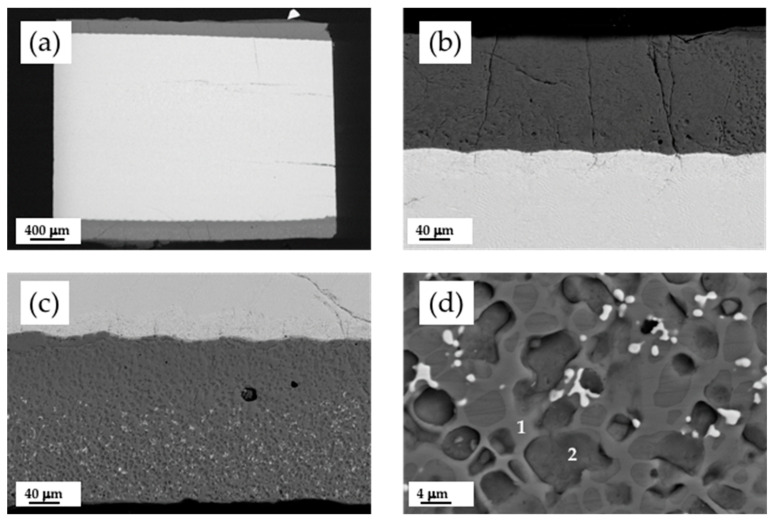
SEM micrographs of a longitudinal cross-section of a cladded eutectic rod (**a**). Detailed views of the coatings (**b**,**c**). Detail of the dendrites of CSZ incorporated into the coating (**d**).

In order to achieve a bioceramic layer covering the CZO-CSZ eutectic cylinder, thicker layers were prepared. The suitable adjustment of the laser power minimized the dilution with the substrate. The bioceramic layer was placed by dip coating on the surface of a CZO-CSZ eutectic rod. For this purpose, ten dips were used at a speed of 3 mm/s. Next, it was sintered at 1200 °C for 12 h, below the melting point, and placed at 1402 °C. This procedure improved the features of the coating in terms of densification and adhesion to the substrate. After this procedure, there was no perceived crack formation or spallation. Finally, the sintered coating was melted by laser. Figure 4a–c show the longitudinal cross-section of a CZO-CSZ eutectic rod laser cladded with a W-TCP coating. Figure 4d corresponds to a detailed view of the coating. The coating showed a clean interface, suggesting good interfacial strength. It was not required to undergo a preheating process during the cladding treatment since the coating did not show visible cracks or delamination. CSZ dendrites of small size were observed within the coating.

**Figure 5:**
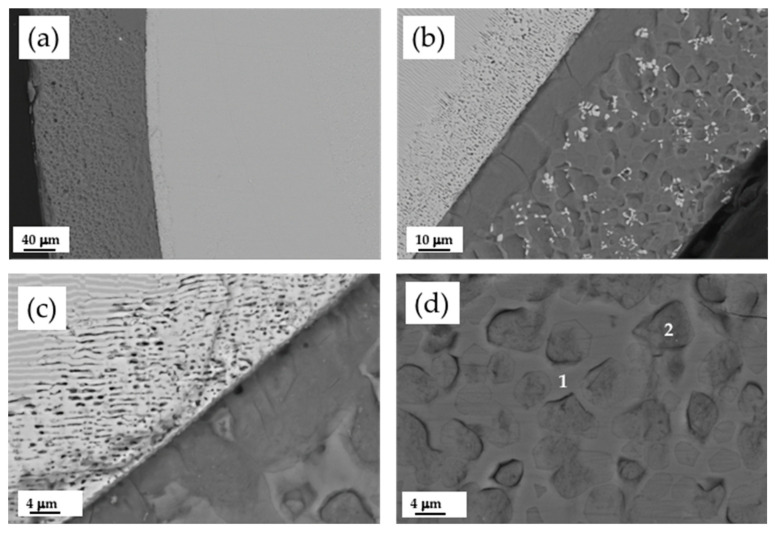
SEM micrographs of a transversal cross-section of a cladded eutectic rod with a bioactive coating (**a**). Detailed view of the coating (**b**). Detailed views of the eutectic substrate/bioactive coating interface and coating (**c**,**d**), respectively.

Figure 5a,b show the transversal cross-section of a W-TCP-coated CZO-CSZ rod. Figure 5c corresponds to a detailed view of the interface. The coating was about 145 µm thick and was formed by two phases. The average composition was near the theoretical one, as seen in Table 3. The theoretical composition of the W-TCP eutectic is shown in parenthesis. The calcium content in the coating is higher and the silicon content is lower than the theoretical ones, which is indicative of the existence of diffusion of these species between the layer and the substrate. Between the substrate and the coating, there is a zone where the CZO phase shows some degradation. The composition of this zone is given in Table 3 and can be compared with the composition of the substrate in an area far from the interface (CZO-CSZ DSEC). Some migration of calcium from the CZO phase to the coating can be detected, as can the incorporation of silicon. The CSZ phase did not undergo any modifications.

When the coating is observed at higher microscope magnifications (Figure 4d and Figure 5d), two phases of similar contrast can be identified. They correspond to silicon calcium phosphate with different contents of phosphorus and silicon, the lighter with a higher content of phosphorus. The relation Ca/(P+Si) is 1.5 and 1.6 for phases 1 and 2, respectively.

### 3.4. Micro-Raman Characterization

**Figure 6:**
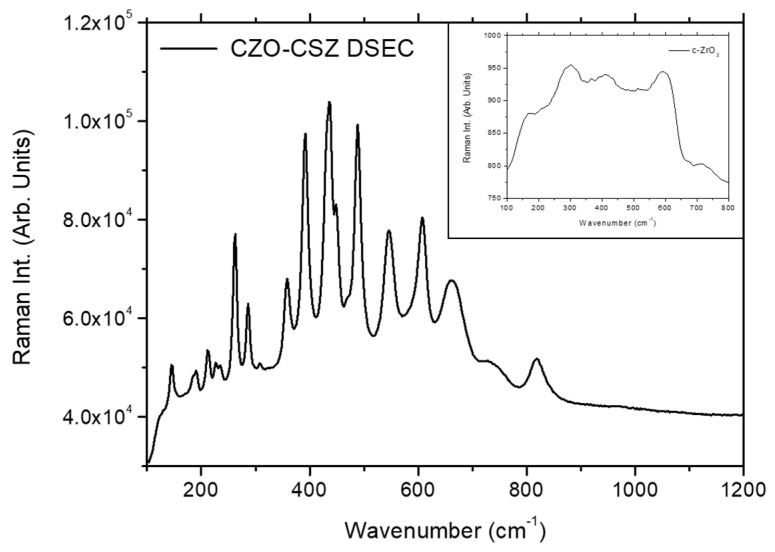
Raman spectra of the CZO-CSZ DSEC and ZrO_2_ cubic phases (inset).

A more precise knowledge of the structure of the DSE CZO-CSZ sample cladded with W-TCP was investigated by micro-Raman assessment. Firstly, µ-Raman characterization was performed in the CZO-CSZ eutectic composite in the 100–1200 cm^−1^ wavenumber region. As shown in Figure 6, the Raman spectra of this eutectic composite presented narrow peaks and broad bands, the positions of which are shown in Table 4. As previously reported, in this eutectic composite, the Raman spectra of the CSZ phase present broad and unresolved bands, whereas the Raman spectra of the CaZrO_3_ phase present highly intense and well-defined peaks [35]. The Raman spectra shown in Figure 6 can be attributed to the convolution of the Raman spectra of both phases. Worth mentioning is the fact that the peaks found at 607 cm^−1^, 660 cm^−1^, and 818 cm^−1^ had not been previously reported yet since, compared to the previous studies carried out on this composite, the wavenumber region in this work was extended up to 1200 cm^−1^. The presence of CSZ in the cubic phase in the eutectic composite before and after soaking in SBF was confirmed by acquiring the Raman signal from the corresponding phase by means of a 0.90 NA objective, as shown in the inset of Figure 6. Neither tetragonal nor monoclinic zirconia was detected, so the cubic structure for this CSZ phase was proven to be independent from soaking.

**Figure 7:**
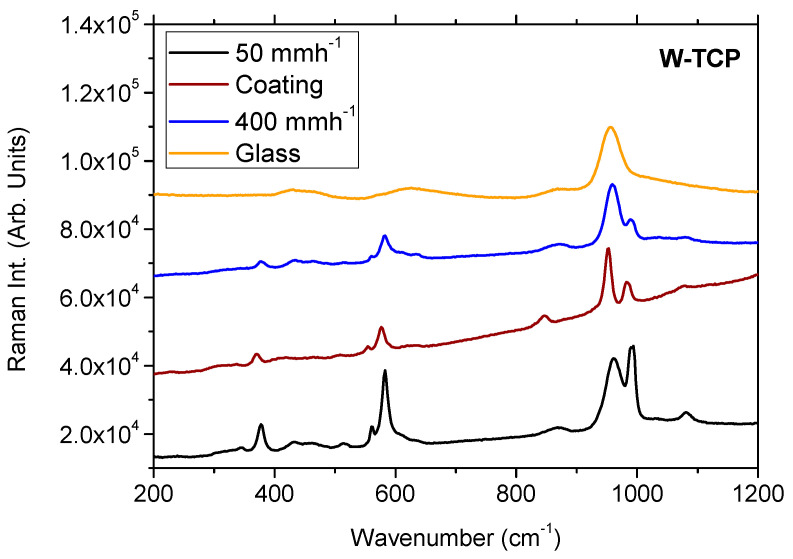
Raman spectra of the W-TCP coating, W-TCP eutectic glass-ceramics grown at 50 mm/h and 400 mm/h, and W-TCP eutectic glass.

Next, Raman characterization was performed on the W-TCP coating. The position of the main peaks and bands presented in the Raman spectra of this layer was significantly different from the ones observed in the CZO-CSZ eutectic composite, principally the peaks placed in the low-frequency range at 953 cm^−1^, 983 cm^−1^, and 1076 cm^−1^ (Figure 7). The position of these peaks is included in Table 4.

Raman characterization also allowed us to confirm the glass-ceramic nature of the W-TCP coating described in the assessment by SEM. Figure 7 compares the Raman spectra of the W-TCP eutectic coating with the ones obtained for W-TCP eutectic glass-ceramics grown at 50 mm/h and 400 mm/h and a W-TCP eutectic glass. In this bioactive eutectic composite, the glassy/crystalline features can be controlled by the growing rate [36,37]. At a low growing rate, the composite is essentially crystalline, whereas as the growing rate increases, the composite is turned into glass. The change in these microstructural features can be observed in the Raman spectra so that the crystalline phase provides a sharp peak at 377 cm^−1^, doublets in the 543–640 cm^−1^ range and in the 920–1020 cm^−1^ range, and broad bands centered at around 870 cm^−1^ and 1079 cm^−1^. As the growing rate increases, the intensity of the low-energy components of both doublets decreases, turning into broad bands for the glass sample. In addition, the broad band at 1079 cm^−1^ also disappears for the glass sample. As shown in Figure 7, the Raman spectra of the W-TCP coating presented features corresponding to a glass-ceramic sample, thus corroborating the findings observed by SEM characterization.

## 4. Conclusions

- The microstructure and mechanical properties of CZO-CSZ DSEC have been studied. Because of their excellent hardness, resistance, toughness, and microstructure stability, they exhibit good characteristics for use as biomaterials.
- Laser alloying and cladding can modify in an effective way CZO-CSZ bioinert eutectics with desired biological properties, making them useful as load-bearing implants for clinical applications.
- The as-formed hybrid material is robust, with excellent adhesion between the coating and the ceramic substrate.
- Raman studies allowed for distinguishing the structure of the CZO-CSZ matrix from the W-TCP coating. Furthermore, the Raman spectra of the coating presented features of a glass-ceramic material.

## Figures and Tables

**Table 1:** Chemical composition of the phases predicted by the phase diagram of CaO-ZrO_2_ for the eutectic composition and experimental chemical composition of the phases for a sample grown at 200 mm/h.

	O	Ca	Zr	O	Ca	Zr
	Theoretical (at%)			Experimental (at%)		
Eutectic	61.54	15.38	23.08	59.94	15.71	24.35
CSZ	63.64	9.09	27.27	61.68	9.87	28.45
CaZrO_3_	60	20	20	58.39	20.93	20.68

**Table 2:** Mechanical properties and phase interspacing of CZO-CSZ eutectic.

Growth Conditions	HV (GPa)	Toughness (Indentation) (MPa m^1/2^)	Flexural Strength (MPa)	Phase Interspacing λ (µm)
300 mm/h	9.13 ± 0.2	2.85 ± 0.24	735 ± 24.46	1.21

**Table 3:** Summary of CZO-CSZ/W-TCP compositions in different zones of the sample.

at. %	O	Ca	Zr	P	Si
Coating average composition	59.6 (60.60)	23.06 (21.21)		6.29 (6.06)	10.46 (12.12)
Phase 1	59.11	24.12		6.84	9.17
Phase 2 globular	61.79	23.76		8.35	6.5
Interface	61.67	9.9	26.59		1.84
CZO-CSZ DSEC	58.45	15.62	25.93		

**Table 4:** Mode frequency for CZO-CSZ, W-TCP coating, W-TCP eutectic glass-ceramics, and W-TCP eutectic glass.

Sample	Frequency (cm^−1^)
CZO-CSZ DSEC	145, 190, 212, 227, 234, 262.5, 286.5, 308, 358, 392, 436, 448, 488, 545.5, 607, 660, 818
W-TCP Eut. Coating	136, 370, 506, 554, 577, 845, 953, 983, 1076
W-TCP Eut. Glass-ceramics	344, 377, 432, 516, 560, 581, 870, 958, 993, 1079
W-TCP Eut. Glass	425, 458, 623, 870, 955

## Data Availability

The data presented in this study are available on request from the corresponding author.
